# Kinetic and thermodynamic insights into sodium ion translocation through the μ-opioid receptor from molecular dynamics and machine learning analysis

**DOI:** 10.1371/journal.pcbi.1006689

**Published:** 2019-01-24

**Authors:** Xiaohu Hu, Yibo Wang, Amanda Hunkele, Davide Provasi, Gavril W. Pasternak, Marta Filizola

**Affiliations:** 1 Department of Pharmacological Sciences, Icahn School of Medicine at Mount Sinai, New York, New York, United States of America; 2 Department of Neurology and Molecular Pharmacology Program, Memorial Sloan Kettering Cancer Center, New York, New York, United States of America; University of Maryland School of Pharmacy, UNITED STATES

## Abstract

The differential modulation of agonist and antagonist binding to opioid receptors (ORs) by sodium (Na^+^) has been known for decades. To shed light on the molecular determinants, thermodynamics, and kinetics of Na^+^ translocation through the μ-OR (MOR), we used a multi-ensemble Markov model framework combining equilibrium and non-equilibrium atomistic molecular dynamics simulations of Na^+^ binding to MOR active or inactive crystal structures embedded in an explicit lipid bilayer. We identify an energetically favorable, continuous ion pathway through the MOR active conformation only, and provide, for the first time: i) estimates of the energy differences and required timescales of Na^+^ translocation in inactive and active MORs, ii) estimates of Na^+^-induced changes to agonist binding validated by radioligand measurements, and iii) testable hypotheses of molecular determinants and correlated motions involved in this translocation, which are likely to play a key role in MOR signaling.

## Author summary

Notwithstanding years of research supporting the notion that μ-opioid receptor (MOR) function can be modulated by sodium ions (Na^+^), a complete understanding of Na^+^ translocation through the receptor and its effect on ligand binding at MOR requires additional information. Here, we use computer simulations to elucidate the energetics involved in sodium binding at inactive and active MOR, the timescales of sodium translocation through these receptor conformations, and the molecular determinants involved in this process.

## Introduction

Evidence of allosteric modulation of receptor signaling by cations was first presented in the literature for opioid receptors (ORs). Specifically, sodium (Na^+^) and lithium, but not other monovalent or divalent cations, were shown to enhance receptor binding of opiate antagonists and to reduce the binding of opiate agonists, thus altering ligand properties *in vivo* [1].

Over the course of years, the original hypothesis that Na^+^ stabilizes an inactive conformation of the receptor was extended to several other G protein-coupled receptors (GPCRs) [2], but it was only recently supported by various ultra-high resolution crystal structures of inactive GPCRs, including that of δ-OR [3]. In this structure, Na^+^ was found to be bound at an allosteric site through coordination with two water molecules as well as receptor residues N131^3.35^, S135^3.39^, and D95^2.50^ (superscripts refer to the Ballesteros-Weinstein generic numbering scheme [4]). Notably, we observed a similar ion coordination in molecular dynamics (MD) simulation studies [5] of Na^+^ binding from the bulk solvent to the inactive μ- and κ-OR (MOR and KOR, respectively) crystal structures embedded in a hydrated 1-palmitoyl-2-oleoyl-sn-glycero-3-phosphocholine (POPC)/10% cholesterol lipid bilayer and at physiological concentrations of Na^+^.

Unlike their inactive crystal structures, experimental structures of active GPCRs (e.g., those of MOR [6, 7]), show a collapsed ion binding site which likely results in weaker Na^+^ binding affinity and consequent ion departure from the receptor. How Na^+^ migrates into the cytosol has recently been described in the literature for the active state of the M2 muscarinic receptor [8]. One conclusion of that study is that Na^+^ egress into the cytosol occurs without significant energy barriers when the D^2.50^ is protonated and another fairly conserved residue, Y^7.53^, is in an upward configuration. Notably, the intracellular egress of Na^+^ is further facilitated by the formation of a hydrated pathway connecting the orthosteric ligand binding pocket to the G protein binding site, a feature that has also been seen in experimental structures (e.g., those of active MOR [6, 7]) as well as in recent simulation studies of GPCRs such as the adenosine A_2A_ receptor [9] and the serotonin 5-HT_1A_ receptor [10].

Herein, using a combination of molecular dynamics (MD), Markov State Models (MSMs), and machine learning tools, we provide, for the first time, estimates of the timescales associated with Na^+^ translocation through the TM helix bundle of either active or inactive MOR conformations embedded in an explicit POPC/10% cholesterol lipid bilayer at a physiological concentration of Na^+^. Moreover, we present complete free-energy profiles of Na^+^ movement through these receptor states, estimates of Na^+^-induced changes to agonist binding validated by radioligand measurements, and testable hypotheses of the most important underlying motions and molecular determinants involved in Na^+^ translocation.

## Results and discussion

To obtain both kinetic and thermodynamic estimates of Na^+^ translocation through active or inactive MOR states, we estimated a Multi-Ensemble Markov Model (MEMM) from a number of umbrella sampling (US) and independent, unbiased MD simulations, using the transition-based reweighted analysis method (TRAM) [11]. Details of this method are reported in the Methods section, while a summary of simulations we have run (S1 Table).

Simulations were run on inactive MOR with the key Na^+^-coordinating residue D114^2.50^ charged, and on active MOR with either a charged or a neutral (protonated) D114^2.50^ for the following reasons: a) the D^2.50^ protonation state is hypothesized to be dependent on the conformational state of the receptor [12], with calculated pKa values above physiological pH and an increased population of the D^2.50^ protonated state in active receptor conformations. While these conclusions were drawn for the β_2_ adrenergic receptor (B2AR), the high degree of structural and sequence conservation of the sodium binding pocket across rhodopsin-like GPCRs [2] suggests transferability to the MOR as well; b) based on calculated pKa values, the proximity of Na^+^ favors a charged D^2.50^ state over the neutral one [13], whereas D^2.50^ likely becomes protonated as Na^+^ moves away from this residue [8]; c) intracellular transfer of sodium is easier when D^2.50^ is protonated [8]; d) ligand-dependent G protein signaling is altered when D^2.50^ is mutated to a neutral amino acid in more than 25 different GPCRs [2]; and e) D^2.50^ is directly involved in Na^+^ coordination in ultra-high resolution crystal structures of inactive but not active GPCRs (e.g., compare inactive DOR [3] with active MOR [6] structures).

The MEMM framework was applied to coordinate trajectories from ~11 μs of combined US and unbiased MD simulations of inactive and active MOR systems. Trajectories obtained for each system were discretized into microstates encoding the Na^+^ position in the receptor, as well as the slowest protein degrees of freedom captured by time-lagged independent component analysis (tICA) of the conformation of protein residues near Na^+^ density values higher than the bulk value in either active or inactive MOR structures (see the Methods section for details of the selection of these residues, S2 Table for a complete list, and S1 Fig for their location in a representative active MOR state from MD).

### Spatial distribution of Na^+^ across the active and inactive MOR

**Fig 1:**
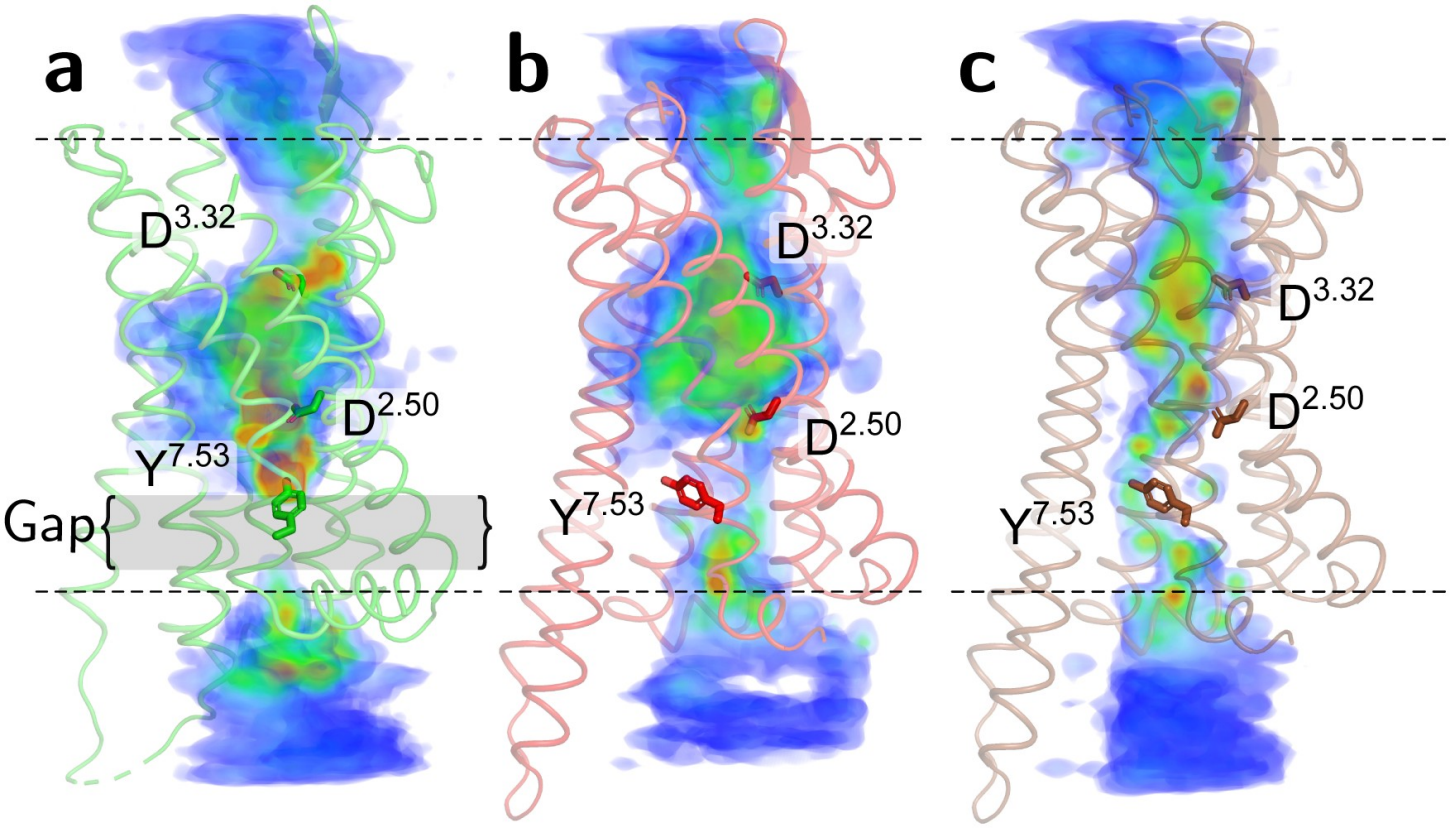
Reweighted Na^+^ density heat maps of simulated active and inactive MOR systems. Residues D147^3.32^, D114^2.50^ and Y336^7.53^ are displayed as sticks to roughly indicate the locations of the orthosteric ligand binding site, the Na^+^ allosteric binding site, and the Na^+^ density gap (in the simulated inactive MOR system only), respectively, in the (a) inactive MOR crystal structure, (b) active MOR crystal structure with charged D^2.50^, and (c) active MOR crystal structure with protonated D^2.50^. Dotted lines symbolize the location of lipid head groups. A blue-to-red color scale illustrates low-to-high Na^+^ densities.

The three-dimensional spatial density distributions of Na^+^ across inactive or active MOR, the latter with either a charged or a protonated D114^2.50^, were obtained from the corresponding combined trajectories of unbiased MD and US simulations (see the Methods section for details). As shown in Fig 1, clear differences exist in the spatial distribution of Na^+^ across the active and inactive MOR, with the inactive receptor structure (Fig 1a) exhibiting several more localized high-density regions for the ion (red hotspots) compared to active MOR (Fig 1b and 1c). Perhaps the most striking difference between the active and inactive MOR is the continuous Na^+^ density distribution observed through the entire TM bundle in the active, but not the inactive, MOR.

### Molecular determinants involved in Na^+^ translocation through inactive and active MOR systems

To generate testable hypotheses of the most important molecular determinants involved in Na^+^ translocation among residues close to high ion densities, we extracted lists of residue pairs (S3 Table) whose minimum heavy atom distance fluctuations had a correlation larger than 0.6 to the most dominant tIC_0_ and tIC_1_ components. Graphs illustrating these selected residue pairs on the inactive or active MOR crystal structures are shown in S2 Fig. These highly correlated inter-residue distance fluctuations to tIC_0_ and tIC_1_ (e.g., those involving the conserved, functionally important, NPxxY(x)_5,6_F motif in rhodopsin-like GPCRs) can be interpreted as the main contributors to the slowest (and most important) motion modes in the simulated inactive and active MOR systems. For instance, out of a total of 176 residue pairs that are highly correlated to tIC_0_ in the simulated inactive MOR (S3 Table), Y336^7.53^, F343^H8^ and N332^7.49^ are involved in 89, 58, and 30 pairs, respectively, while other residues are only involved in 3 or fewer pairs. Notably, disruption of the interaction between rhodopsin residues corresponding to positions Y336^7.53^ and F343^H8^ in MOR has been shown to lead to rhodopsin activation [14].

Overall, the simulated active MOR systems exhibited a reduced number of highly correlated inter-residue distance fluctuations to the most dominant tIC_0_ and tIC_1_ components compared to the inactive simulated MOR (S3 Table), although most of them still involved residues of the NPxxY(x)_5,6_F motif. While N332^7.49^ also stood out as a main contributor to the most important motions of the two simulated active MOR systems, Y336^7.53^ and F343^H8^ were found not to be involved in the most important motions in the active MOR system with a protonated D114^2.50^. Notably, recently published MD simulations of three different rhodopsin-like GPCRs [9] revealed that three distinct rotamer conformations of the conserved Y^7.53^ residue were correlated with the occlusion or opening of a continuous intrinsic water channel characteristic of an inactive or active conformational state of the receptor, respectively. The observed larger number of relevant tIC_0_ and tIC_1_ components involving Y336^7.53^ in the inactive MOR compared to the active receptor (S3 Table) suggests that Na^+^ translocation to the cytosol in inactive MOR is hindered by several residues that have to move in a concerted manner to enable Y336^7.53^ to change its rotameric state and allow opening of the continuous intrinsic water channel.

The highly correlated inter-residue distance fluctuations to tIC_1_ are very different in the simulated inactive and active MOR systems (S3 Table). While in the inactive MOR all highly-correlated pairs to tIC_1_ involve the conserved F289^6.44^ residue in concerted motion with TM1, TM2, and TM3 residues, in the active MOR simulated with a charged D114^2.50^, roughly half of the pairs are between residues located in the orthosteric ligand binding pocket (e.g., Y148^3.33^, Y149^3.34^, and N150^3.35^) and residues at the allosteric Na^+^ binding site (e.g., A113^2.49^ and D114^2.50^). In contrast, in the active MOR simulated with a protonated D114^2.50^, all but one highly-correlated pairs to tIC_1_ involve the so-called “rotamer toggle switch” W293^6.48^ in concerted motion with TM3, TM4, TM5, and TM7 residues. Notably, many of these residues have a known functional role [15], which, based on the above, might be due to their contribution to Na^+^ translocation.

### Thermodynamics of Na^+^ translocation through both the inactive and active MOR

**Fig 2:**
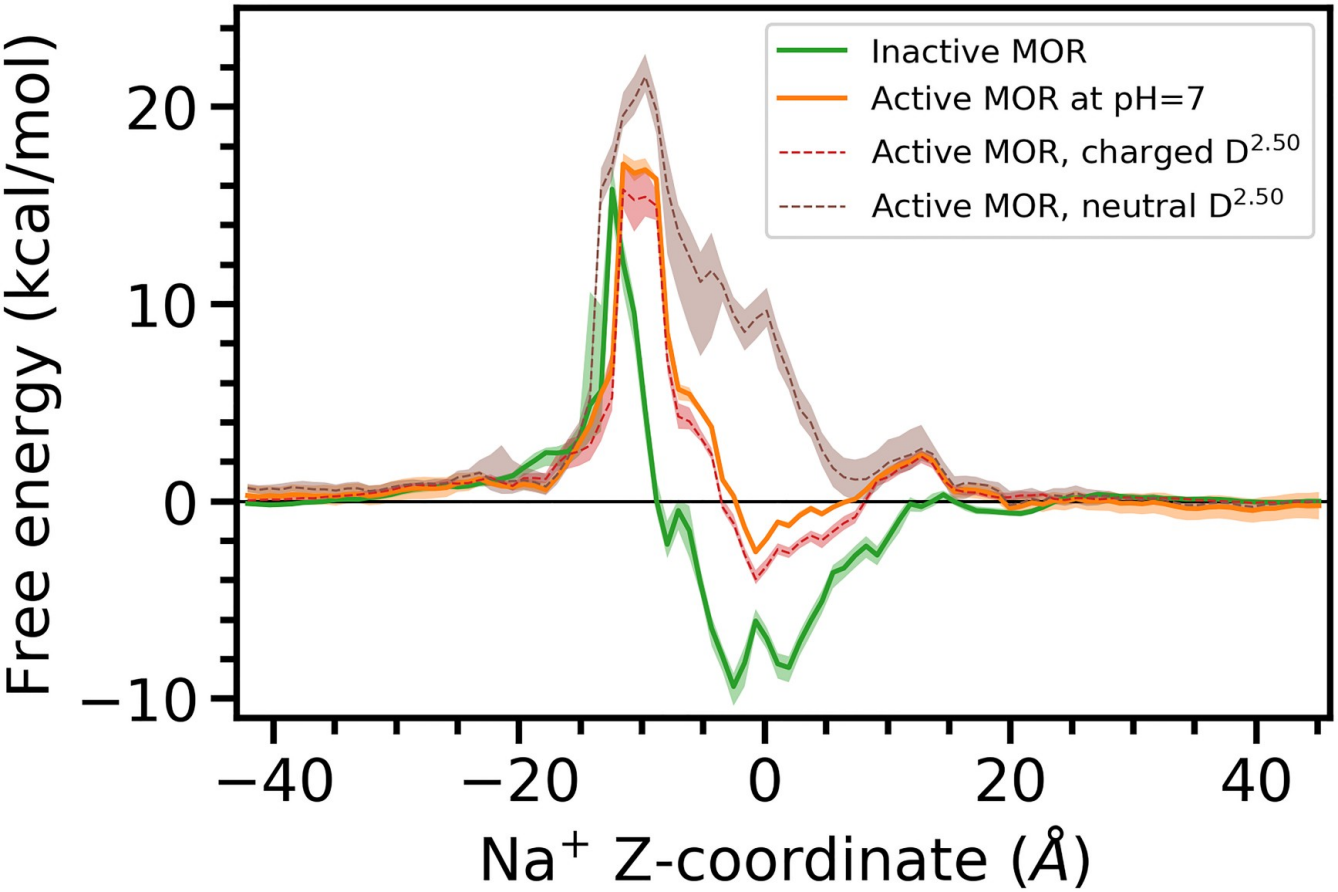
Integrated free energy profiles of Na+ translocation through inactive MOR (green) and active MOR with either charged or protonated D114^2.50^ (red and brown, respectively), as well as at pH = 7 (orange), as a function of the Na^+^ z-coordinate. The errors of the free energy are obtained via a bootstrapping procedure as described in the Methods.

Using the MEMM framework (see Methods), we calculated the thermodynamics of Na^+^ translocation through both the inactive and active MOR. The derived free-energy profiles of inactive and active MOR are reported in Fig 2.

As seen in this figure, Na^+^ binding at its allosteric site (Na^+^ *z*-coordinate = 0) is ~6 kcal/mol more energetically favorable in the inactive MOR compared to the receptor active state at pH = 7 (up to ~11 kcal/mol compared to active MOR with a neutral D^2.50^). This means that in the inactive MOR, the Na^+^ ion needs to overcome a significantly higher free-energy barrier to egress to the cytosol compared to the active MOR (especially the state with a neutral D^2.50^, where the free energy barrier for the translocation is the lowest), making ion egress less likely to occur in an inactive MOR structure.

### Kinetics of Na^+^ translocation through both the inactive and active MOR

**Fig 3:**
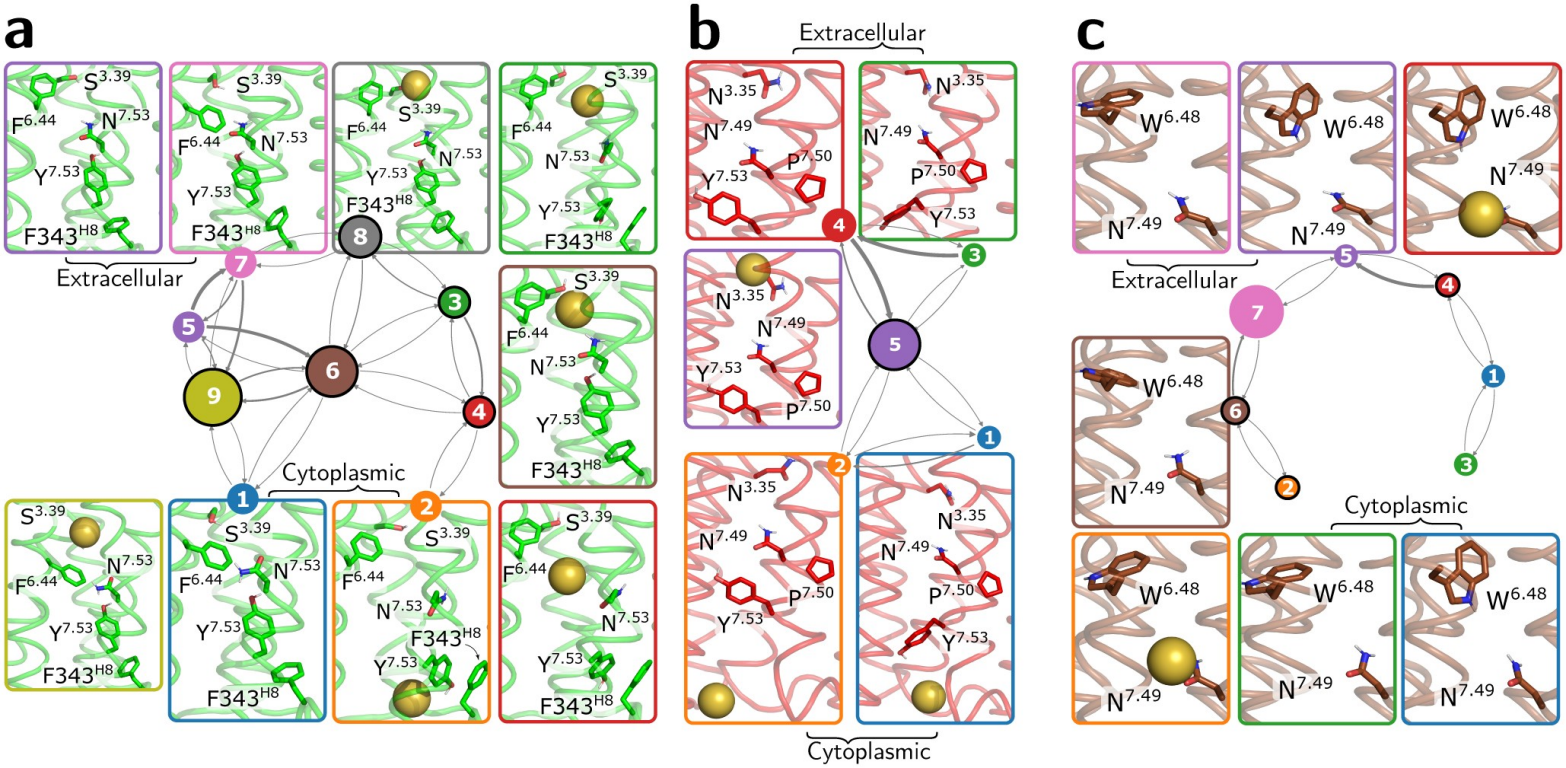
Transition networks formed by the metastable states obtained from the Markov state models constructed for the inactive MOR (panel a) and active MOR with charged or protonated D114^2.50^ (panels b and c, respectively). States are labeled according to the Na^+^ position relative to the receptor as extracellular or cytoplasmic states, while bound states are highlighted with a black contour. The size of the circles depicting the metastable states is proportional to the probability of individual states and the thickness of the arrows connecting them is proportional to the transition flux between the states. Three-dimensional structures are shown in insets for each metastable state and represent the center of the corresponding PCCA cluster. Residues highlighted as sticks are the highly coupled residues from tICA with the most significant sidechain conformational change among different PCCA cluster center structures based on visual inspection.

We also built MSMs to elucidate the kinetics of Na^+^ translocation in the inactive or active MOR. For this, we not only present the results of MOR simulated with either a charged or protonated D114^2.50^, but also those of a mixed model system at pH 7 that would capture, in principle, protonation changes of the residue (see Methods for details). Details of the MEMM construction and its validation are provided in the Methods section and S3 Fig, respectively. The microstates of the Markov model for each system were divided into a small set of metastable states, which were labeled according to the Na^+^ position relative to the receptor as “extracellular”, “bound”, or “cytoplasmic” states. The transition networks between these states are shown in Fig 3a for the inactive MOR, and in Fig 3b and 3c for active MOR with charged and protonated D114^2.50^, respectively. S4, S5 and S6 Tables report the transition times between these metastable states for inactive MOR, active MOR with charged D114^2.50^, and active MOR with protonated D114^2.50^, respectively. The observed larger number of Na^+^ bound states in the inactive MOR (Fig 3a) indicates a more rugged energy landscape than in the active receptor with multiple local minima that can trap the Na^+^ ion.

**Fig 4:**
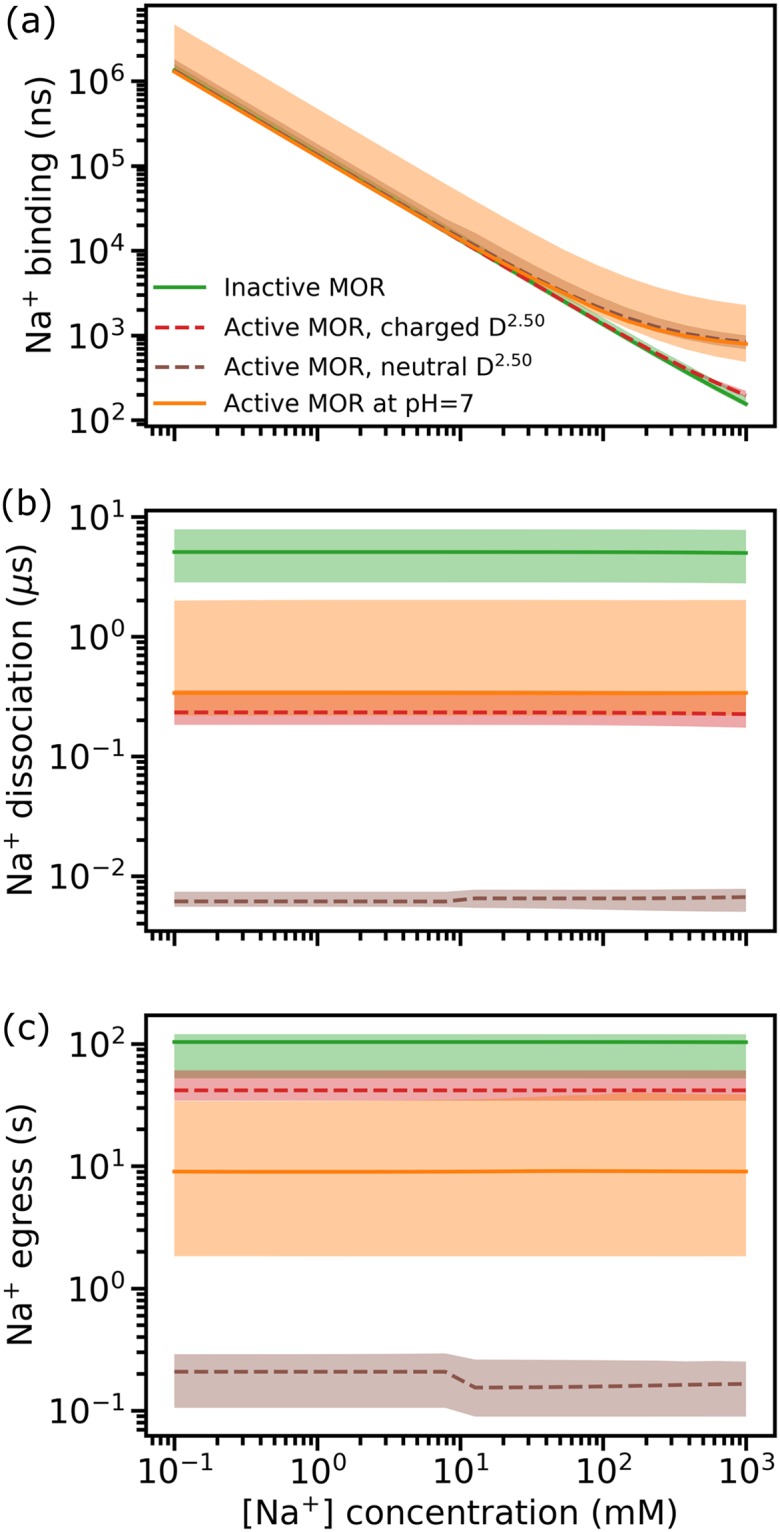
Estimated transition times of (a) Na^+^ binding (from extracellular to bound states, in ns), (b) Na^+^ dissociation (from bound to extracellular states, in μs), and (c) Na^+^ egress (from bound to intracellular states, in s) as a function of extracellular Na^+^ concentration. The Na^+^ concentration in the cytoplasm is assumed to be constant. Transition times are calculated as the median of the mean first passage times calculated from individual bootstrap samples and the full sample. Confidence intervals were estimated as the differences from the 1^st^ and 3^rd^ quartiles.

In order to obtain kinetic estimates of Na^+^ translocation that can be compared to experiments, we coupled the Markov model obtained from TRAM to a bulk state with a fixed ion concentration, and calculated Na^+^ transition times from extracellular bulk to bound states (Na^+^ binding; Fig 4a), from bound to extracellular states (Na^+^ dissociation; Fig 4b), and from bound to intracellular states (Na^+^ egress; Fig 4c) at different concentrations (see the Methods section).

As expected, the Na^+^ binding kinetics is highly concentration-dependent while the Na^+^ dissociation and egress kinetics are virtually independent on concentration. While the timescale of Na^+^ binding to the receptor is similar for the different receptor conformations, the timescales of Na^+^ dissociation and egress differ between inactive and active MOR. Specifically, based on the “mixed model” at pH 7, Na^+^ dissociation from the active MOR is estimated to take ~0.3 (0.2, 2.0) μs, which is significantly faster than in the inactive MOR (~5.5 (3.0, 8.0) μs). This is consistent with the prediction that a Na^+^ bound state in the active MOR has significantly higher free energy compared to the inactive MOR. The Na^+^ egress timescales are estimated to be of the order of hundreds of milliseconds to ~60 s depending on whether the active MOR has a protonated or charged D114^2.50^, whereas Na^+^ leaves the inactive MOR state in ~100 seconds, suggesting that Na^+^ can more easily migrate to the cytosol in an active MOR with a protonated D114^2.50^ than in the inactive MOR. Notably, these predicted timescales are similar to the experimentally derived lifetimes of GPCR/G protein complexes [16, 17]. From the concentration-dependent MSM model, we estimated the Na^+^ binding affinity to be 23 mM (from 14 mM to 50 mM with errors) at a ligand-free, inactive MOR or significantly lower (850 mM; from 650 mM to 1.3 M with errors) at active MOR models with charged D^2.50^ (S4 Fig). The corresponding values for the active models with a neutral D^2.50^ and the model at a constant pH = 7.0 are in excess of 1 M.

### Na^+^ modulation of ligand binding to MOR

**Fig 5:**
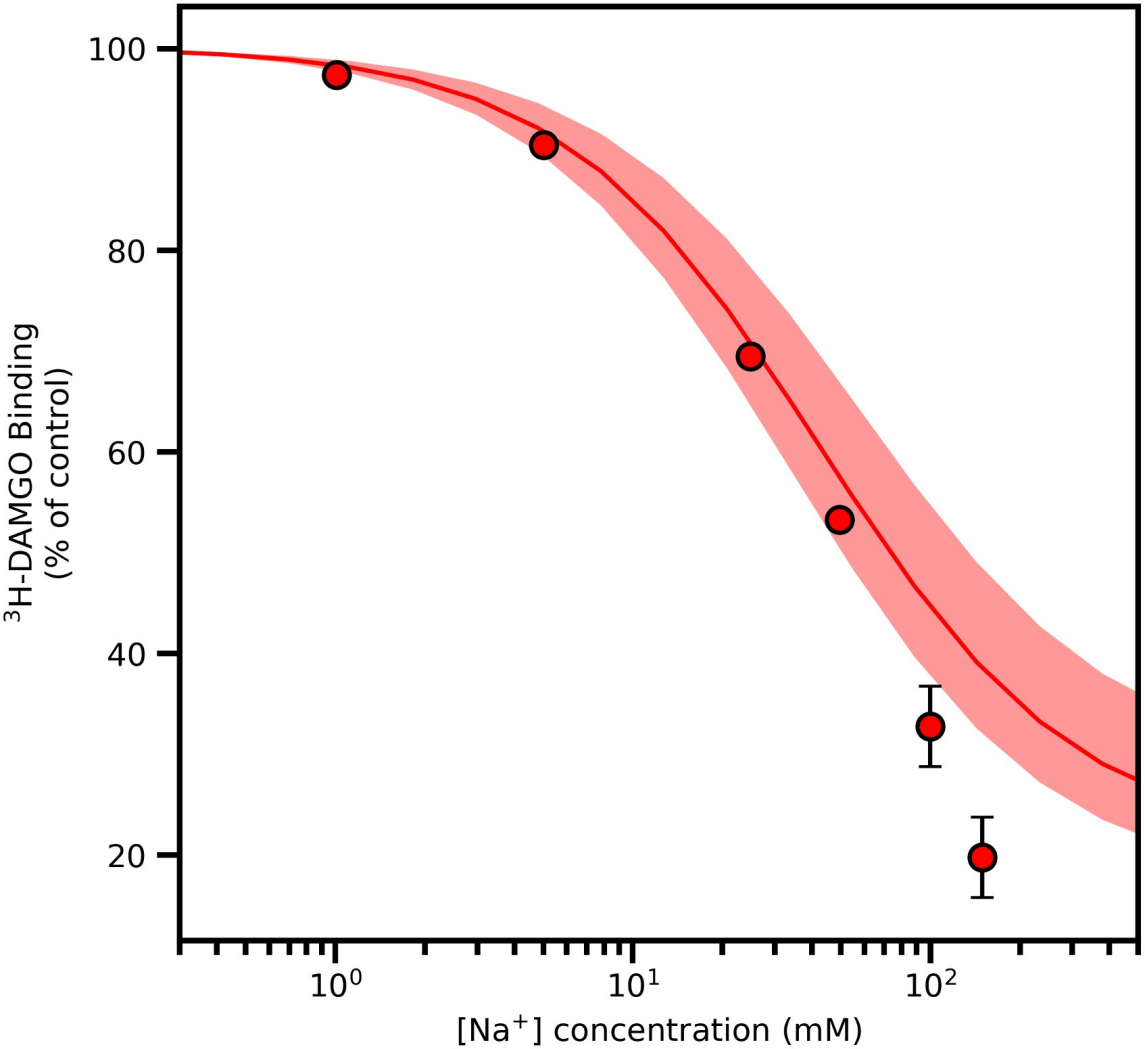
Percent change in [^3^H]-DAMGO binding as a function of increasing concentrations of NaCl, reported for a concentration of [^3^H]-DAMGO of 1 nM, and assuming a [^3^H]-DAMGO binding constant of 4 nM. Circles represent values from radioligand binding experiments, the solid line represents the predictions from the simulation data while the shaded region corresponds to the errors associated with the predictions. Binding values are the means ± s.e.m. of three independent experiments. Error bars that are not visible are smaller than the size of the symbol.

To explain Na^+^ modulation of ligand binding to MOR, we applied the two-state receptor theory (see Methods), and used the free-energies obtained from the aforementioned models to calculate sodium-induced stabilization of the receptor inactive state relative to the active one. Given that antagonists bind with equal affinity to both active and inactive conformations of the receptor, this model is consistent with the observation that antagonist bound fractions are not affected by ion concentrations. In the case of a full agonist, we can quantitatively estimate the extent to which its bound fraction is modulated by the ion concentration. Specifically, assuming a binding affinity of ~4 nM [18] for MOR agonist DAMGO at the active conformation of the receptor, we estimated the relative bound fraction of DAMGO to active MOR at constant pH = 7 as a function of the ion concentration as described in the Methods section, and report these results in Fig 5, together with experimental values obtained by radioligand binding experiments.

The calculated percent reduction in agonist binding at increasing ion concentrations (solid line in Fig 5) by this simple model is in good agreement with the experimental data, and indicates that ligand binding modulation is triggered by the stabilization of the inactive conformation of the receptor in the presence of sodium, which affects orthosteric ligand binding affinity. The model estimates that 60 mM of Na^+^ (with a confidence interval between 50 and 150 mM) are required to achieve a 50% reduction of DAMGO binding, in excellent agreement with the experimental value of 60 mM. For sodium concentrations above 200 mM, the simple model employed here slightly underestimates the effect of sodium, suggesting that direct interactions with the ligand or double occupation of the receptor by multiple ions might play a role at high ionic strength. Saturation experiments (see S5 Fig) are also in reasonable agreement with this simple allosteric model. In the presence of sodium (25 mM), the affinity of ^3^H-DAMGO does not appreciably change (K_D_ 1.8 and 0.89 nM for control and sodium conditions) while the B_max_ is lowered by 50% (333 fmol/mg protein to 156 fmol/mg protein). This is consistent with a shift of the receptor to an inactive state from an active one.

In summary, the combination of MD, MSMs, and machine learning tools is powerful in that it provides, for the first time, both kinetic and thermodynamic estimates of Na^+^ translocation through active or inactive MOR states in a membrane mimetic environment and at a physiological concentration of Na^+^. The results provide quantitative support to the notion that Na^+^ can more easily egress from the cytosol in an active MOR with a protonated D114^2.50^ than in an inactive receptor, as well as testable hypotheses of the most important underlying motions and molecular determinants involved in Na^+^ translocation.

## Methods

### MD simulation details and systems setup

The active and inactive MOR systems were modeled based on the respective crystal structures (PDB entries 5C1M and 4DKL, respectively). The missing loop between TM5 and TM6 in 4DKL was added as described previously [14, 19]. For the active MOR, the N-terminal region preceding residue M65 was removed and the missing residues on the helix 8 at the C-terminus was rebuilt using the Prime package included in the Schrödinger’s suite [20] to ensure both simulated systems had identical primary sequences. Both inactive and active MOR models were embedded in a POPC and cholesterol bilayer with a mixing POPC:cholesterol≈9:1 ratio and an area of 79×79 Å^2^. The membrane and protein were then neutralized and solvated with explicit TIP3P water and a NaCl concentration of 150 mM. The entire simulation systems contained approximately 60,000 atoms with a volume of 79×79×106 Å^3^ and were assembled using the CHARMM-GUI webserver [21]. The CHARMM36 force field [22, 23] was used to model protein, lipids and ions and all molecular dynamics (MD) simulations were carried out using the NAMD software package [24]. Both inactive and active MOR systems were simulated in the NPT ensemble using the Nosé-Hoover Langevin piston method [25, 26] to maintain the pressure at 1 atm, and a Langevin thermostat to maintain the temperature around 310 K. Long-range electrostatic interactions were calculated using the Particle-Mesh Ewald (PME) algorithm [27]. The van der Waals interactions were switched off gradually between 10 and 12 Å. Periodic boundary conditions were applied to the simulation boxes, and an integration time step of 2 fs was used for all simulations. After a multi-step equilibration with gradually decreasing harmonic constraints on lipid and protein heavy atoms, following the CHARMM-GUI membrane builder equilibration protocol, an additional 60 ns unconstrained equilibration run was carried out. The last snapshot of this equilibration run was used as a starting point for umbrella sampling simulations.

### Umbrella sampling and unbiased simulations

US simulations were carried out to enhance sampling of the Na^+^ ion translocation across the membrane through the interior of the TM helix bundle of MOR. A bias was applied to the Z-coordinate of a reference sodium ion, parallel to the normal vector of the membrane surface and measured from a reference position (*Z = 0*) corresponding to the location of the Na^+^ allosteric binding site defined as the center of mass of the C_α_ atoms of residues D114^2.50^, N150^3.35^, W293^6.48^, and Y326^7.43^ (the superscripts refer to the Ballesteros-Weinstein numbering scheme [4]). For each active and inactive MOR system, 157 starting configurations for US windows, uniformly spaced by 0.5 Å, were selected to cover the entire TM region of the protein and part of the bulk solvent (from Z = +40 Å in the extracellular region to Z = -35 Å in the intracellular region). The reference sodium ion was slowly pulled from one window to another. A harmonic biasing potential with a force constant of 10 kcal/(mol·Å^2^) was applied along the Z variable to constrain the Na^+^ ion in the center of each window. A flat-bottom cylindrical constraint with radius of 15 Å was applied to avoid insufficient sampling of the reference ion in the bulk solvent and to prevent the disturbance by other ions. To prevent the drift of MOR in the membrane, a harmonic potential was applied to the head groups of POPC lipids with a force constant of 10 kcal/(mol·Å^2^). Each US window was run for at least 7 ns (in addition to 1 ns of equilibration run) or until the relative entropy [28] reached values below 0.2 for an average simulation length of 11.8 ns and a maximum simulation length of 100 ns. To assess the kinetic behavior of the ion across the protein, we also carried out a set of unbiased simulations, starting from the last frame of each umbrella sampling window and running additional 12 ns of simulation. The same simulation settings as the biased simulations were used, while all restraint potentials on the sodium and the lipids were removed.

### Three-dimensional sodium density distributions

3D density distribution maps were built using a grid-based approach. First, global translational and rotational motions of the protein in all simulation trajectories were removed by fitting to a reference structure using the protein C_α_ atoms root mean square deviation (RMSD). Then, a 3D rectangular grid covering the entire TM domain of the protein and a small part of the bulk solvent was built using a uniform grid spacing of 1.25 Å in all directions and amounting to a volume of 30×30×70 Å^3^, and a total of 32,256 grid points. The 3D bins defined by the grid were used to obtain the reweighted probability for the reference ion’s 3D position using the data from both umbrella sampling and unbiased simulations and the weighted histogram analysis method (WHAM) estimator implemented in the python library PyEmma [29]. The reweighted density values were normalized to the averaged density values of the grid points in the bulk solvent (0.01 particles/nm^3^). The results were saved as a dx grid file, which was subsequently rendered as layered 3D color maps using the visualization software Pymol [30].

### Selection of input features for the time-lagged independent component analysis (tICA)

Sodium-interacting protein residues across the TM bundle were selected using the active MOR with charged D114^2.50^ as a reference structure, which has sodium density registered over a larger area compared to the three simulated MOR systems. A total of 105 residues within 4.4 Å from density grid points with values at least 7 times larger than the bulk region density were identified using an in-house python code.

The pair-wise distances between residue heavy atoms as a function of time were extracted using PyEmma and used as input features for tICA [31]. tICA uses a linear transformation to map the original input data ***r***(*t*) onto a new set of time-lagged independent components (see reference [31] for details). These components are correlated and their autocorrelation is maximal at a fixed lag-time [31]. Notably, the most dominant components span a linear subspace that contains the slowest, and therefore most relevant, degrees of freedom. These components can therefore provide the dimensional reduction that is necessary for the construction of a MSM [31, 32] using PyEmma. A lag time of 0.1 ns was used for our tICA calculations and the first two most dominant independent components, tIC_0_ and tIC_1_, were used to describe the protein dynamics portion of our final MSM, which includes the Na^+^ motions as well.

The residue pairs whose minimum heavy atom distance fluctuations exhibited a larger than 0.6 correlation (the absolute value of the Pearson correlation coefficient) relative to tIC_0_ and tIC_1_ were considered the slowest (and most important) motion modes.

### Construction and clustering of the tICA free energy landscapes

We projected trajectories of the inter-residue minimum distance fluctuations between heavy atoms of the 105 selected residues near Na^+^ high-density regions onto the two most dominant independent components tICA_0_ and tICA_1_ with PyEmma and calculated the free energy landscapes of all three simulated MOR systems i.e., active MOR with either charged or protonated D114^2.50^, and the inactive MOR. We included tICs extracted from both unbiased MD simulations and umbrella sampling to construct the combined free energy landscapes sampled in both sets of simulations. The combined landscapes were then subjected to k-means clustering using PyEmma. Different k values were selected depending on the complexity of the individual free-energy landscape. Specifically, we chose k = 5, 7 and 4 for the inactive MOR, active MOR with charged D114^2.50^, and active MOR with protonated D114^2.50^ MOR, respectively. The resulting cluster centers were then used to assign the frames in trajectories from the unbiased and umbrella samplings simulations individually.

### Thermodynamic and kinetic estimates from multi-ensemble Markov model

In order to optimally utilize both sets of unbiased MD and umbrella sampling simulations to derive equilibrium and kinetic properties of the system, we used the recently published transition-based reweighing method (TRAM) [11] to estimate a MEMM. This approach aims at overcoming the limitations of standard MD simulations (e.g., insufficient sampling of transition states) by integrating the results of enhanced sampling techniques such as umbrella samplings [11, 33].

Since the Na^+^ binding, dissociation and egress from the TM bundle does not only depend on the Na^+^ movement alone, but also on the protein conformational dynamics, we designed a MEMM that takes both aspects into account. The trajectories from both the biased and unbiased simulations were discretized into microstates encoding the Na^+^ position, as well as the slowest protein degrees of freedom captured by tICA, which are represented by the transitions between the conformational states on the free energy landscape in the space of tIC_0_ and tIC_1_ approximated by the k-mean clusters of the landscape. Specifically, microstates were defined based on (i) the z coordinate of the Na^+^ ion, which was divided into 100 bins covering the entire range of the umbrella sampling, and (ii) *N* k-means clustering of the two slowest tICA_0_ and tICA_1_ components, leading to a total of *N*×100 microstates, with *N* = 6, 5, and 4 for the inactive MOR, active MOR with charged D114^2.50^, and active MOR with protonated D114^2.50^, respectively. We label the microstates as (*z*, *i*), with 1 ≤ *z* ≤ 100 and 1 ≤ *i* ≤ *N*. The discretized trajectories were used together to obtain a maximum-likelihood TRAM estimation of the transition matrix in the unbiased thermodynamic state via the python package PyEmma [29]. A lag time of 200 frames (or 0.4 ns), selected based on the convergence of the implied time scales, was used for TRAM.

The free-energy of the microstates $\overset{-}{G}(z,i)$ was obtained from the steady-state probabilities from the Markov model estimated from TRAM. In order to obtain the one-dimensional free energy profiles of the active and inactive MOR systems as a function of *z*, we integrated out the tICA dimensions via the relation:

$$G(z)=-k_{B}Tln[\sum_{i=1}^{N}exp\;(-\frac{\overset{-}{G}(z,i)}{k_{B}T})]$$ (1)

Next, we calculated the timescales employed by the reference sodium ion to bind to and dissociate from the extracellular side of the receptor, as well as to egress from the cytoplasmic side. Considering the much higher Na^+^ concentrations in the extracellular region compared to the cytoplasmic side of the cell membrane under physiological conditions and the resulting unfavorable membrane potential, no ion binding from the intracellular side was taken into account.

For the kinetics estimates, sodium trajectories that crossed the periodic boundary between two unit cells along the z-direction were split in order to remove artificial transitions between microstates close to the intracellular side and the extracellular side (i.e. z~0 and z~100, respectively) without actually going through the receptor.

A kinetic model was constructed by first assigning all microstates from the MSM estimated from TRAM into a small number of metastable states (*N*_pcca_ = 9, 5 and 7 for inactive, active with charged D^2.50^ and active with neutral D^2.50^ MORs, respectively) by using the Perron-cluster cluster analysis [34] (PCCA+). A *N*_pcca_ × *N*_pcca_ transition matrix between the *N*_pcca_ metastable states was then estimated using the Hummer-Szabo method [35], and a Markov model estimated from this transition matrix using the PyEMMA package.

Furthermore, metastable states were clustered into three groups depending on whether Na^+^, occupied the intracellular, bound, or cytoplasmic regions, respectively. Specifically, microstate (*i*, *z*) was assigned to the cytoplasmic state if 1 ≤ *z* ≤ 10. A microstate belonging to one of the bins with 30 ≤ *z* ≤ 60 was considered to belong to the ion bound state, whereas microstates with 90 ≤ *z* ≤ 100 were considered to belong to the extracellular state. Each of the *N*_pcca_ metastable states was then assigned to one of the three groups (intracellular, bound, or cytoplasmic) if 90% of its microstates belonged to such a group.

### Constant pH kinetic model for the active receptor

In order to assess the effect of D^2.50^ protonation on sodium binding, we constructed a kinetic model that combines the properties of the sodium binding to the receptor with charged and neutral D^2.50^, which we label with indices *α* and *β*, respectively. Specifically, to establish a common reference state for the active model of MOR, we assumed that the pKa of D^2.50^ in the absence of Na^+^ nearby is *pK*_*a*_ ≈ 9 [13], which corresponds to a free-energy difference of Δ*G*_0_ ≈ 2.6 kcal/mol at physiological *pH* ≈ 7.0. Using this shift, we expressed the free energy of the microstates *z* of the MOR system with charged D^2.50^ as

$$\varepsilon_{\alpha }^{\left(\text{Act}.\right)}(z)=e_{\alpha }^{\left(\text{Act}.\right)}(z)+{\Delta G}_{0}$$ (2)

where $e_{\alpha }^{\left(\text{Act}.\right)}(z)$ are the free-energies obtained from the simulation trajectories of the MOR system with charged D^2.50^. We then obtained the thermodynamic properties for the combined system as a function of the ion position as

$$\text{exp}\;\left(-\frac{\varepsilon^{\left(\text{Act}.\right)}(z)}{k_{B}T}\right)=\text{exp}\;(-\frac{e_{\alpha }^{\left(\text{Act}.\right)}(z)+\;{\Delta G}_{0}}{k_{B}T})+\text{exp}\;\left(-\frac{e_{\beta }^{\left(\text{Act}.\right)}(z)}{k_{B}T}\right)$$ (3)

where $e_{\beta }^{\left(\text{Act}.\right)}(z)$ are the free-energies obtained from simulation trajectories of the MOR system with neutral D^2.50^, while the probability of observing a charged sidechain as a function of the ion position is:

$$\begin{array}{cccc} p_{\alpha }\left(z\right) & =\frac{1}{1+\text{exp}\left(-\frac{e_{\beta }^{\left(\text{Act}.\right)}\left(z\right)-e_{\alpha }^{\left(\text{Act}.\right)}\left(z\right)-\;\Delta G_{0}}{k_{B}T}\right)} & =\frac{1}{1+\frac{\pi_{\beta }^{\left(\text{Act}.\right)}\left(z\right)}{\pi_{\alpha }^{\left(\text{Act}.\right)}\left(z\right)}\text{exp}\left(\frac{\;\Delta G_{0}}{k_{B}T}\right)}\equiv & \text{exp}\left(-\frac{\Delta G\left(z\right)}{k_{B}T}\right) \end{array}$$ (4)

We then modeled the rates for a fixed protonation state of D^2.50^ using the kinetic models obtained from analysis of the simulation run on MOR with either a charged or neutral D^2.50^:

$$K(z,i,x;z^{\prime },i^{\prime },x)=K_{x}(z,i;z^{\prime },i^{\prime })$$ (5)

where *x* = *α*, *β* (i.e., charged and neutral D^2.50^ states, respectively), while *z* and *i* indicate, as before, the position of the sodium ion and the conformational microstate of the protein. Following the evidence from NMR [36] we modeled the protonation process for given *z* and *i* with a constant deprotonation rate (off-rate) *k*_off_

$$K(z,i,\beta ;z^{\prime },i^{\prime },\alpha )\;=\;\delta (z,z^{\prime })\delta (i,i^{\prime })\;k_{\text{off}}$$ (6)

Based on published work [36], we used *k*_off_ = 10^6^ *s*^−1^. The protonation state that ensures that the free energy difference between the two protonation states is preserved is therefore

$$K(z,i,\alpha ;z^{\prime },i^{\prime },\beta )\;=\;\delta (z,z^{\prime })\delta (i,i^{\prime })\;k_{\text{off}}exp(\frac{\Delta G(z)}{k_{B}T})$$ (7)

while *δ*(*z*, *z*′)*δ*(*i*, *i*′) guarantees that only protonation events for a fixed ion position and side-chain conformations are possible. Finally, the rates between the PCCA macrostates *a* and *b* defined above were approximated as:

$$K(a,x;b,x\prime )\;=\;\sum_{j,z\prime \in b}\sum_{i,z\in a}\pi_{x}(i,z)K(z,i,x;z^{\prime },i^{\prime },x\prime )$$ (8)

The matrix *K* was used to calculate kinetic rates for the constant pH = 7.0 model of the active MOR system.

### Kinetics at physiological sodium concentrations

In order to address the effects of sodium at physiological concentrations, we supplemented the Markov model by coupling it to reference states corresponding to the intracellular and extracellular bulk with constant sodium concentrations [Na^+^]_IC_ and [Na^+^]_EC_, respectively. We modeled the kinetics of ions across the receptor stepwise [37], as follows:

$${\text{Na}}_{\text{EC}}+R\;\begin{array}{c} k_{\text{EC}}^{+}\\ ⇌\\ k_{\text{EC}}^{-} \end{array}{\left(\text{Na}∙R\right)}_{\text{EC}}\begin{array}{c} k_{ij}\\ ⇌\\ k_{ji} \end{array}\text{Na}\;R\begin{array}{c} k_{jl}\\ ⇌\\ k_{lj} \end{array}{\left(\text{Na}∙R\right)}_{\text{IC}}\begin{array}{c} k_{\text{IC}}^{-}\\ ⇌\\ k_{\text{IC}}^{+} \end{array}{\text{Na}}_{\text{IC}}+R$$ (9)

where Na_EC_ and Na_IC_ indicate a cation in the extracellular or intracellular space, respectively and parenthesis indicate the formation of an encounter complex, defined as the presence of an ion within a cylinder of radius *r*_0_ = 1.5 nm in the extracellular or intracellular region of the bulk. Rates *k*_*ab*_ were obtained from the estimated Markov model, while the rates for the formation of the encounter complexes, $k_{\text{EC}}^{+}$ and $k_{\text{IC}}^{+}$, were obtained from the 3D Smoluchowski expressions for a given ion diffusion constant *D*_Na_ ≅ 20 nm^2^/*μs* and bulk concentration,

$$k_{\text{EC}}^{+}=4\pi D_{\text{Na}}{r_{\text{EC}}[{\text{Na}}^{+}]}_{\text{EC}}$$ (10)

where *r*_EC_ is encounter complex radius. The rates of ion dissociation from the encounter complex, $k_{\text{EC}}^{-}$ and $k_{\text{IC}}^{-}$ determine the capture probabilities, *γ*_EC_ and *γ*_IC_ defined as the probability of an ion to take part in the binding reaction, conditional on having formed the encounter complex:

$$\gamma_{\text{EC}}=\frac{k_{\text{EC}}^{-}}{k_{\text{EC}}^{-}+\sum_{j}k_{ij}}$$ (11)

A similar equation for *γ*_IC_ was defined for the intracellular encounter complex. The values of *γ* were estimated [38] from the unbiased simulations described in the text. The Na^+^ binding, dissociation, and egress rates were calculated by coarse-graining the transition matrix corresponding to the stepwise kinetic model and defined, respectively, as the rate of transition between the extracellular unbound and the bound state, between the bound and the extracellular unbound state, and between the bound and the intracellular unbound state.

### Allosteric effects of ion binding

We employed a minimal two-state model for receptor activation, which resulted in the same functional form as the operational Black-Leff model. Let *τ*_*u*_ and *τ*_*b*_ be the equilibrium constants between the active and inactive states of the ligand-free and ligand-bound receptors, respectively, and let *K* and *K*^⋆^ be the binding affinities of the ligand to the inactive and active receptor states, respectively. Then the fraction of receptors bound to a *ligand* is:

$$f_{b}=\frac{\left[L\right]}{L_{50}+[L]}$$ (12)

where

$$L_{50}=K^{⋆}\frac{1+\tau_{u}}{1+\tau_{b}}$$ (13)

Notably, for antagonists, *τ*_*u*_ ∼ *τ*_*b*_, and *L*_50_ does not depend on the equilibrium. For full agonists, on the other hand, *τ*_*b*_ ≪ *τ*_*u*_ and therefore *L*_50_ ≅ *K*^⋆^(1 + *τ*_*u*_). Thus, the *percent change* of the fraction of bound ligands at ligand concentration $x=[L]/L_{50}^{\left(0\right)}$ when the sodium concentration changes from 0 to [Na^+^] is:

$$\frac{\Delta f_{b}}{f_{b}}\;=\;\frac{f_{b}(\left[{\text{Na}}^{+}\right])-f_{b}(\text{0})}{f_{b}(\text{0})}\;=\;\frac{1-\rho (\left[{\text{Na}}^{+}\right])}{x+\rho (\left[{\text{Na}}^{+}\right])}$$ (14)

where $\rho (\left[{\text{Na}}^{+}\right])=L_{50}^{\left([{\text{Na}}^{+}]\right)}/L_{50}^{\left(0\right)}$. While varying concentrations of sodium can also affect the affinity of ligands (*K*^⋆^), we posit, in agreement with the well-established assumption in the literature (see, e.g. [2] and references therein), that the dominant mechanism of modulation was through changes in the stability of the active and inactive states of the receptor (*τ*_*u*_):

$$\rho (\left[{\text{Na}}^{+}\right])≃\frac{\tau_{u}(\left[{\text{Na}}^{+}\right])}{\tau_{u}^{0}}$$ (15)

and we used the results from our simulations to estimate this ratio. Specifically, the kinetic models obtained from the TRAM estimation characterized the dynamics of two states of the receptor. If we denote with $p_{i}^{\left(\text{Ina}.\right)}$ and with $p_{i}^{\left(\text{Act}.\right)}$ the probabilities obtained for the steady-state of the two models, we can express the relative free-energies of all the states of the receptor as

$$\{\begin{array}{c} \varepsilon_{i}^{\left(\text{Ina}.\right)}=-\;\text{log}\;p_{i}^{\left(\text{Ina}.\right)}\\ \varepsilon_{i}^{\left(\text{Act}.\right)}=-\;\text{log}\;p_{i}^{\left(\text{Act}.\right)}-\mu \end{array}$$ (16)

where we measure energies in units of the thermal energy *kT* and *μ* is the activation free-energy of the receptor in the absence of sodium. Specifically, the fraction of receptors in the activated MOR state is

$$f^{⋆}\;=\;\frac{\sum_{i}e^{-\varepsilon_{i}^{\left(\text{Act}.\right)}}}{\sum_{i}e^{-\varepsilon_{i}^{\left(\text{Act}.\right)}}+\sum_{i}e^{-\varepsilon_{i}^{\left(\text{Ina}.\right)}}}\;=\;\frac{e^{-\mu }}{e^{-\mu }+Z^{\left(\text{Ina}.\right)}{/Z}^{\left(\text{Act}.\right)}}$$ (17)

where we indicated with *Z*^(Ina.)^ and *Z*^(Act.)^ the partition functions obtained summing over all the states of the respective systems. Thus

$$\tau_{u}=\frac{1-f^{⋆}}{f^{⋆}}=\frac{Z^{\left(\text{Ina}.\right)}}{e^{-\mu }Z^{\left(\text{Act}.\right)}}$$ (18)

which gives in the end:

$$\rho (\left[{\text{Na}}^{+}\right])={\left(\frac{Z^{\left(\text{Ina}.\right)}}{Z^{\left(\text{Act}.\right)}}\right)}_{\left[{\text{Na}}^{+}\right]}{\left(\frac{Z^{\left(\text{Ina}.\right)}}{Z^{\left(\text{Act}.\right)}}\right)}_{0}^{-1}$$ (19)

### Error estimations with bootstrapping

A bootstrapping procedure similar to the one described previously [11] was used to estimate the errors of free energy and transition times. For each simulation system, 12 bootstrap samples were obtained randomly selecting (with repetitions) unbiased trajectories for a total number of frames equal to 90% of the full sample. The resampled unbiased trajectories and the full set of umbrella sampling trajectories were then combined and used to construct individual MEMMs via TRAM. The transition times were estimated as the median of the mean first passage times calculated from individual bootstrap samples and the full sample. Confidence intervals were estimated as the differences from the 1^st^ and 3^rd^ quartiles.

### Binding experiments

Binding studies were carried out using membranes from CHO cells stably transfected with the MOR-1 clone, as previously described [39]. Membranes (200 μg protein) were prepared and binding assays carried out with ^3^H-DAMGO incubated at 25°C for 1.5 h in potassium phosphate buffer (50 mM, pH 7.4) with MgSO_4_ (5 mM) in a volume of 500 μl. At the end of the incubation, the samples were filtered over glass fiber filters and binding determined by scintillation counting. In each experiment, samples were assayed in triplicate and specific binding defined as the difference between total binding and binding in the presence of levallorphan (10 μM). Studies looking at the effects of varying concentrations of NaCl utilized the indicated concentration of NaCl with ^3^H-DAMGO (1 nM). Values are the average of three independent replications of each experiment.

## Supporting information

S1 Table Summary of number, type, and length of simulations. (DOCX)

S2 Table List of mouse MOR residues selected for tICA analysis. (DOCX)

S3 Table List of MOR residue pairs whose minimum heavy atom distance fluctuation has a correlation larger than 0.6 to the most dominant tICA_0_ and tICA_1_ components in the simulated active or inactive MOR systems. (DOCX)

S4 Table Transition times (in μs) between metastable states obtained from the MSM constructed for the simulated inactive MOR system. The label “Inf” indicates the absence of transitions between two states.
(DOCX)

S5 Table Transition times (in μs) between metastable states obtained from the MSM constructed for the simulated active MOR system with a charged D^2.50^. The label “Inf” indicates the absence of transitions between two states.
(DOCX)

S6 Table Transition times (in μs) between metastable states obtained from the MSM constructed for the simulated active MOR system with a protonated D^2.50^. The label “Inf” indicates the absence of transitions between two states.
(DOCX)

S1 Fig Spatial location of the 105 residues listed in S1 Table in a representative active MOR state from molecular dynamics simulations shown in vertical and horizontal views. The Cα atoms of these residues are shown as spheres.
(TIFF)

S2 Fig Graphical illustration of MOR residue pairs whose minimum heavy atom distance fluctuations have a correlation larger than 0.6 to the most dominant tIC_0_ and tIC_1_ components. (TIFF)

S3 Fig The MSM implied time scales as a function of lag-time for the first 10 eigenvalues obtained for each simulated MOR system. The data points correspond to the median estimated from all bootstraps samples and the full sample. The upper and lower error bars represent the differences between the median and 1^st^ and 3^rd^ quantiles, respectively.
(TIFF)

S4 Fig Fraction of Na^+^-bound receptor as a function of the extracellular sodium concentration for the inactive MOR (green), active MOR with charged D114^2.50^ (red), active MOR with protonated D114^2.50^ (brown), and active MOR at constant pH = 7 (orange). A dashed black line indicates 50% occupation probability that intersects the curves at the corresponding binding affinity values. The errors obtained from the bootstrap samples are shown as transparent colored bands around the lines.
(TIFF)

S5 Fig Saturation of ^3^H-DAMGO binding in the absence and presence of NaCl. Binding of ^3^H-DAMGO was carried out with for increasing ligand concentrations in the absence (red) and presence (blue) of NaCl (25 nM). Results are the means ± s.e.m. of three independent replications. Nonlinear regression analysis of the curves is indicated by the solid lines. K_D_ values for control and NaCl groups (1.78 and 0.87 nM, respectively) were similar while Bmax values revealed a decrease in the number of sites of 50% (333 fmol/mg protein to 156 fmol/mg proteins).
(TIFF)
