# Supplementary material for: Kinetic and thermodynamic insights into sodium ion translocation through the μ-opioid receptor from molecular dynamics and machine learning analysis
Source: PLoS Comput Biol. 2019 Jan 24;15(1):e1006689. doi: 10.1371/journal.pcbi.1006689 (PMC6363219; doi:10.1371/journal.pcbi.1006689)
Supplement: S1 Table — (DOCX) [file pcbi.1006689.s001.docx]

| System | Unbiased MD simulations | Umbrella sampling simulations | Total |
| --- | --- | --- | --- |
| Inactive MOR | 159 independent trajectories, 1.90 μs | 159 umbrella windows, 2.67 μs | 4.57 μs |
| Active MOR with charged D^2.50^ | 159 independent trajectories, 1.90 μs | 159 umbrella windows, 1.04 μs | 2.94 μs |
| Active MOR with protonated D^2.50^ | 159 independent trajectories, 1.90 μs | 159 umbrella windows, 1.90 μs | 3.80 μs |
| Total | 5.70 μs | 5.61 μs | 11.31 μs |
