# Supplementary material for: Kinetic and thermodynamic insights into sodium ion translocation through the μ-opioid receptor from molecular dynamics and machine learning analysis
Source: PLoS Comput Biol. 2019 Jan 24;15(1):e1006689. doi: 10.1371/journal.pcbi.1006689 (PMC6363219; doi:10.1371/journal.pcbi.1006689)
Supplement: S2 Table — (DOCX) [file pcbi.1006689.s002.docx]

| Domain | Residues |
| --- | --- |
| TM1 | Y75^1.39^, V78^1.42^, C79^1.43^, G82^1.46^, N86^1.50^, I93^1.57^, Y96^1.60^ |
| ICL1 | T 97^ICL1^ |
| TM2 | T101^2.37^, T103^2.39^, N104^2.40^, Y106^2.42^, I107^2.43^, N109^2.45^, L110^2.46^, A111^2.47^, L112^2.48^, A113^2.49^, D114^2.50^, A115^2.51^, L116^2.52^, A117^2.53^, T118^2.54^, S119^2.55^, T120^2.56^, L121^2.57^, Q124^2.60^, N127^2.63^, G131^2.67^ |
| ECL1 | T132^ECL1^, W133^ECL1^ |
| TM3 | I144^3.29^, I146^3.31^, D147^3.32^, Y148^3.33^, Y149^3.34^, N150^3.35^, M151^3.36^, F152^3.37^, T153^3.38^, S154^3.39^, I155^3.40^, F156^3.41^, T157^3.42^, L158^3.43^, C159^3.44^, T160^3.45^, M161^3.46^, S162^3.47^, D164^3.49^, R165^3.50^ |
| ICL2 | R179^ICL2^ |
| TM4 | N191^4.49^, I198^4.56^ |
| ECL2 | R211^ECL2^, Q212^ECL2^, G213^ECL2^, S214^ECL2^, I215^ECL2^, D216^ECL2^, C217^ECL2^, T218^ECL2^, L219^ECL2^, T220^ECL2^ |
| TM5 | V236^5.42^, A240^5.46^, P244^5.50^, Y252^5.58^ |
| TM6 | I278^6.33^, M281^6.36^, V285^6.40^, A287^6.42^, V288^6.43^, F289^6.44^, I290^6.45^, V291^6.46^, C292^6.47^, W293^6.48^, T294^6.49^, I296^6.51^, H297^6.52^ |
| ECL3 | E310(ECL3) |
| TM7 | T315^7.32^, W318^7.35^, H319^7.36^, C321^7.38^, I322^7.39^, A323^7.40^, L324^7.41^, G325^7.42^, Y326^7.43^, T327^7.44^, N328^7.45^, S329^7.46^, C330^7.47^, L331^7.48^, N332^7.49^, P333^7.50^, L335^7.52^, Y336^7.53^, A337^7.54^, D340^7.57^, E341^7.58^ |
| H8 | N342^H8^, F343^H8^ |
