# Supplementary material for: Kinetic and thermodynamic insights into sodium ion translocation through the μ-opioid receptor from molecular dynamics and machine learning analysis
Source: PLoS Comput Biol. 2019 Jan 24;15(1):e1006689. doi: 10.1371/journal.pcbi.1006689 (PMC6363219; doi:10.1371/journal.pcbi.1006689)
Supplement: S3 Table — (DOCX) [file pcbi.1006689.s003.docx]

| System | component |  |
| --- | --- | --- |

| Active MOR with charged D^2.50^ | tIC_0_ (73 pairs) | N332-N86, N332-A111, N332-L112, N332-A115, N332-V236, N332-A240, N332-P244, N332-A287, N332-I290, N332-W293, N332-T294, N332-H297, P333-S162, P333-R179, L335-Y106, L335-I107, L335-N109, L335-L110, L335-F156, L335-T157, L335-C159, L335-T160, L335-M161, L335-S162, L335-D164, L335-R165, L335-R179, L335-P244, L335-Y252, L335-I278, L335-V285, Y336-Y75, Y336-V78, Y336-Y106, Y336-I107, Y336-L110, Y336-L121, Y336-G131, Y336-F156, Y336-T157, Y336-L158, Y336-C159, Y336-T160, Y336-M161, Y336-S162, Y336-D164, Y336-R179, Y336-N191, Y336-P244, Y336-Y252, Y336-V288, Y336-C292, Y336-T315, Y336-W318, Y336-H319, Y336-C321, Y336-I322, Y336-A323, Y336-L324, Y336-Y326, Y336-T327, Y336-N328, Y336-S329, Y336-L331, A337-F289, A337-I290, A337-H319, A337-A323, A337-L324, A337-T327, A337-L331, D340-P333, F343-N332 |
| --- | --- | --- |
|  | tIC_1_ (17 pairs) | Y148-A113, Y148-D114, Y149-A113, Y149-D114, Y149-A117, Y149-T118, N150-N86, N150-A113, N150-D114, N150-W133, S329-Y149, C330-Y149, L331-Y149, N332-Y149, P333-Y149, A337-Y148, A337-Y149 |
| Active MOR with protonated D^2.50^ | tIC_0_ (5 pairs) | N332-L112, N332-A240, N332-F289, L335-A111, L335-L112 |
|  | tIC_1_ (9 pairs) | W293-F152, W293-N191, W293-I198, W293-A240, W293-P244, Y326-W293, N328-T294, N332-W293, P333-W293 |
| Inactive MOR | tIC_0_ (176 pairs) | N332-I93, N332-T103, N332-N104, N332-Y106, N332-I107, N332-N109, N332-A111, N332-L112, N332-D147, N332-S154, N332-D164, N332-R179, N332-V236, N332-A240, N332-P244, N332-A287, N332-V288, N332-F289, N332-I290, N332-V291, N332-C292, N332-W293, N332-T294, N332-I296, N332-H297, N332-W318, N332-C321, N332-I322, N332-G325, Y336-Y75, Y336-V78, Y336-C79, Y336-G82, Y336-N86, Y336-I93, Y336-Y96, Y336-T97, Y336-T101, Y336-T103, Y336-N104, Y336-Y106, Y336-N109, Y336-L110, Y336-A111, Y336-L112, Y336-A113, Y336-D114, Y336-A115, Y336-L116, Y336-A117, Y336-T118, Y336-S119, Y336-T120, Y336-L121, Y336-Q124, Y336-N127, Y336-G131, Y336-T132, Y336-W133, Y336-I144, Y336-I146, Y336-D147, Y336-Y148, Y336-Y149, Y336-N150, Y336-M151, Y336-F152, Y336-T153, Y336-S154, Y336-I155, Y336-F156, Y336-T157, Y336-L158, Y336-C159, Y336-T160, Y336-M161, Y336-S162, Y336-D164, Y336-R165, Y336-R179, Y336-N191, Y336-I198, Y336-R211, Y336-Q212, Y336-G213, Y336-S214, Y336-I215, Y336-D216, Y336-C217, Y336-T218, Y336-L219, Y336-T220, Y336-V236, Y336-A240, Y336-P244, Y336-Y252, Y336-V285, Y336-A287, Y336-F289, Y336-I290, Y336-V291, Y336-C292, Y336-W293, Y336-T294, Y336-I296, Y336-H297, Y336-E310, Y336-T315, Y336-W318, Y336-H319, Y336-C321, Y336-I322, Y336-A323, Y336-L324, Y336-G325, Y336-Y326, Y336-N328, Y336-S329, F343-Y75, F343-V78, F343-C79, F343-G82, F343-N86, F343-I93, F343-T101, F343-T103, F343-N104, F343-Y106, F343-I107, F343-N109, F343-L110, F343-T118, F343-S119, F343-T120, F343-L121, F343-N127, F343-G131, F343-T132, F343-M151, F343-F152, F343-T153, F343-S154, F343-I155, F343-F156, F343-T157, F343-L158, F343-C159, F343-T160, F343-M161, F343-S162, F343-D164, F343-R165, F343-R179, F343-N191, F343-I198, F343-V236, F343-A240, F343-P244, F343-Y252, F343-I278, F343-M281, F343-V285, F343-A287, F343-F289, F343-I290, F343-T315, F343-H319, F343-I322, F343-A323, F343-Y326, F343-T327, F343-S329, F343-C330, F343-L331, F343-N332, F343-P333 |
|  | tIC_1_ (6 pairs) | F289-N86, F289-Y96, F289-I107, F289-Y148, F289-M151, F289-F152 |
