# Supplementary material for: Kinetic and thermodynamic insights into sodium ion translocation through the μ-opioid receptor from molecular dynamics and machine learning analysis
Source: PLoS Comput Biol. 2019 Jan 24;15(1):e1006689. doi: 10.1371/journal.pcbi.1006689 (PMC6363219; doi:10.1371/journal.pcbi.1006689)
Supplement: S4 Table — The label “Inf” indicates the absence of transitions between two states. (DOCX) [file pcbi.1006689.s004.docx]

| **Metastable states** | **1** | **2** | **3** | **4** | **5** | **6** | **7** | **8** | **9** |
| --- | --- | --- | --- | --- | --- | --- | --- | --- | --- |
| **1** | 4.0e-4 | 2.8e10 | 2.7e7 | 2.1e7 | Inf | 5.4e-1 | Inf | Inf | 8.8e-1 |
| **2** | 1.2e11 | 4.0e-4 | Inf | 3.0e-1 | 1.5e5 | Inf | 1.0e7 | 1.5e1 | Inf |
| **3** | 1.2e9 | Inf | 4.0e-4 | 2.7e-2 | Inf | 3.5e0 | 5.1e3 | 6.6e-2 | Inf |
| **4** | 4.7e9 | 1.4e1 | 1.3e-1 | 4.0e-4 | Inf | 9.4e-1 | 3.0e3 | Inf | Inf |
| **5** | Inf | 6.3e10 | Inf | Inf | 4.3e-4 | 1.2e-2 | 1.0e-2 | Inf | 7.3e-2 |
| **6** | 2.7e8 | Inf | 3.9e7 | 2.1e6 | 3.4e0 | 4.0e-4 | Inf | 1.6e-1 | 2.4e-2 |
| **7** | Inf | 1.4e13 | 6.6e8 | 8.1e7 | 3.4e-2 | Inf | 4.1e-4 | 5.4e0 | 1.9e-2 |
| **8** | Inf | 8.5e8 | 3.6e5 | Inf | Inf | 8.2e-2 | 2.3e-2 | 4.0e-4 | Inf |
| **9** | 6,4e8 | Inf | Inf | Inf | 2.9e1 | 3.5e-2 | 2.4e0 | Inf | 4.0e-4 |
