# Supplementary material for: Kinetic and thermodynamic insights into sodium ion translocation through the μ-opioid receptor from molecular dynamics and machine learning analysis
Source: PLoS Comput Biol. 2019 Jan 24;15(1):e1006689. doi: 10.1371/journal.pcbi.1006689 (PMC6363219; doi:10.1371/journal.pcbi.1006689)
Supplement: S5 Table — The label “Inf” indicates the absence of transitions between two states. (DOCX) [file pcbi.1006689.s005.docx]

| **Metastable states** | **1** | **2** | **3** | **4** | **5** |
| --- | --- | --- | --- | --- | --- |
| **1** | 4.0e-4 | 4.1e-1 | 2.8e6 | Inf | 4.7e2 |
| **2** | 2.4e0 | 4.0e-4 | 9.2e5 | Inf | 1.5e2 |
| **3** | 3.8e10 | 2.0e9 | 4.0e-4 | 6.9e-2 | 2.2e0 |
| **4** | Inf | Inf | 2.6e0 | 4.0e-4 | 5.0e-2 |
| **5** | 8.1e8 | 4.4e7 | 2.9e2 | 1.6e-1 | 4.0e-4 |
