# Supplementary material for: Kinetic and thermodynamic insights into sodium ion translocation through the μ-opioid receptor from molecular dynamics and machine learning analysis
Source: PLoS Comput Biol. 2019 Jan 24;15(1):e1006689. doi: 10.1371/journal.pcbi.1006689 (PMC6363219; doi:10.1371/journal.pcbi.1006689)
Supplement: S6 Table — The label “Inf” indicates the absence of transitions between two states. (DOCX) [file pcbi.1006689.s006.docx]

| **Metastable states** | **1** | **2** | **3** | **4** | **5** | **6** | **7** |
| --- | --- | --- | --- | --- | --- | --- | --- |
| **1** | 4.0e-4 | Inf | 1.6e-1 | 9.1e2 | Inf | Inf | 1.5e5 |
| **2** | Inf | 4.0e-4 | Inf | 1.9e4 | 1.2e5 | 3.3e0 | Inf |
| **3** | 1.3e0 | Inf | 4.0e-4 | 2.9e5 | Inf | 2.5e9 | 3.4e7 |
| **4** | 1.9e-1 | 9.4e-1 | 7.8e0 | 5.0e-4 | 1.9e-3 | 3.3e0 | Inf |
| **5** | Inf | 1.9e8 | Inf | 6.3e4 | 4.0e-4 | Inf | 2.5e-1 |
| **6** | Inf | 1.3e5 | 7.2e13 | 2.7e9 | Inf | 4.3e-4 | 4.4e-3 |
| **7** | 3.0e11 | Inf | 8.4e12 | Inf | 7.4e1 | 5.0e-2 | 4.0e-4 |
