# Supplementary figures and images for: Kinetic and thermodynamic insights into sodium ion translocation through the μ-opioid receptor from molecular dynamics and machine learning analysis
Source: PLoS Comput Biol. 2019 Jan 24;15(1):e1006689. doi: 10.1371/journal.pcbi.1006689 (PMC6363219; doi:10.1371/journal.pcbi.1006689)

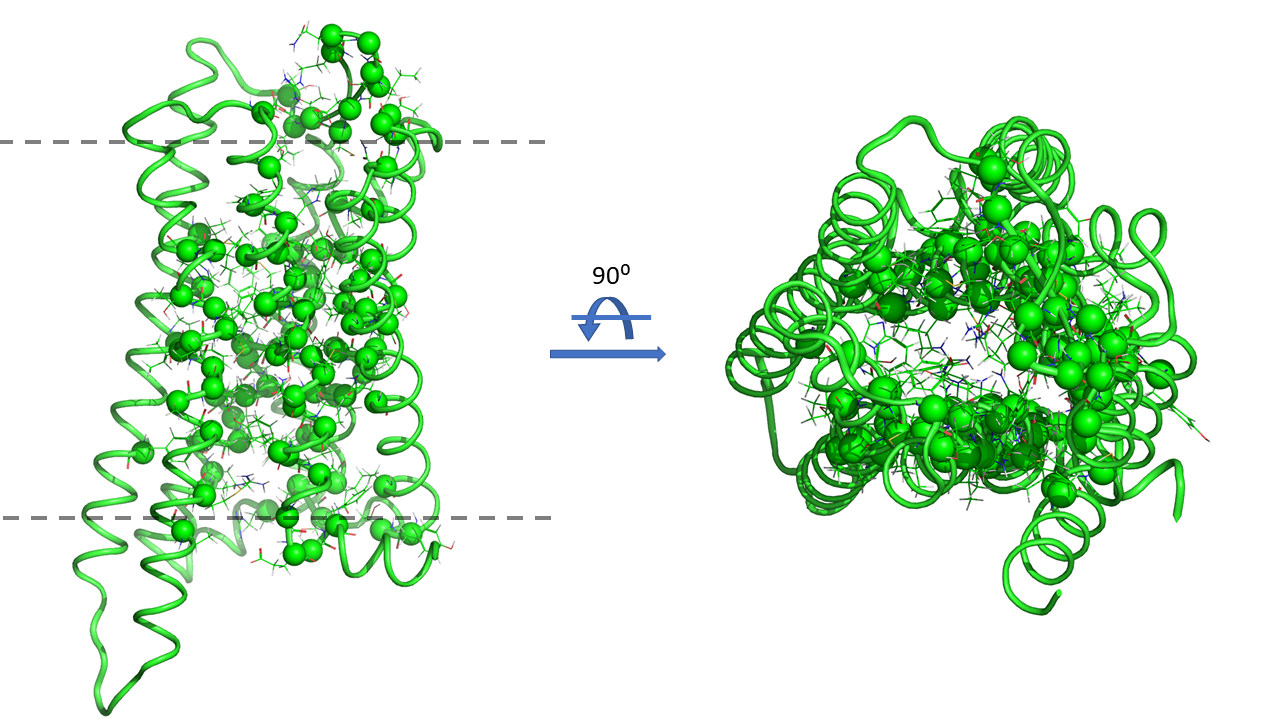

Supplement: S1 Fig — The Cα atoms of these residues are shown as spheres. (TIFF) [file pcbi.1006689.s007.tiff]

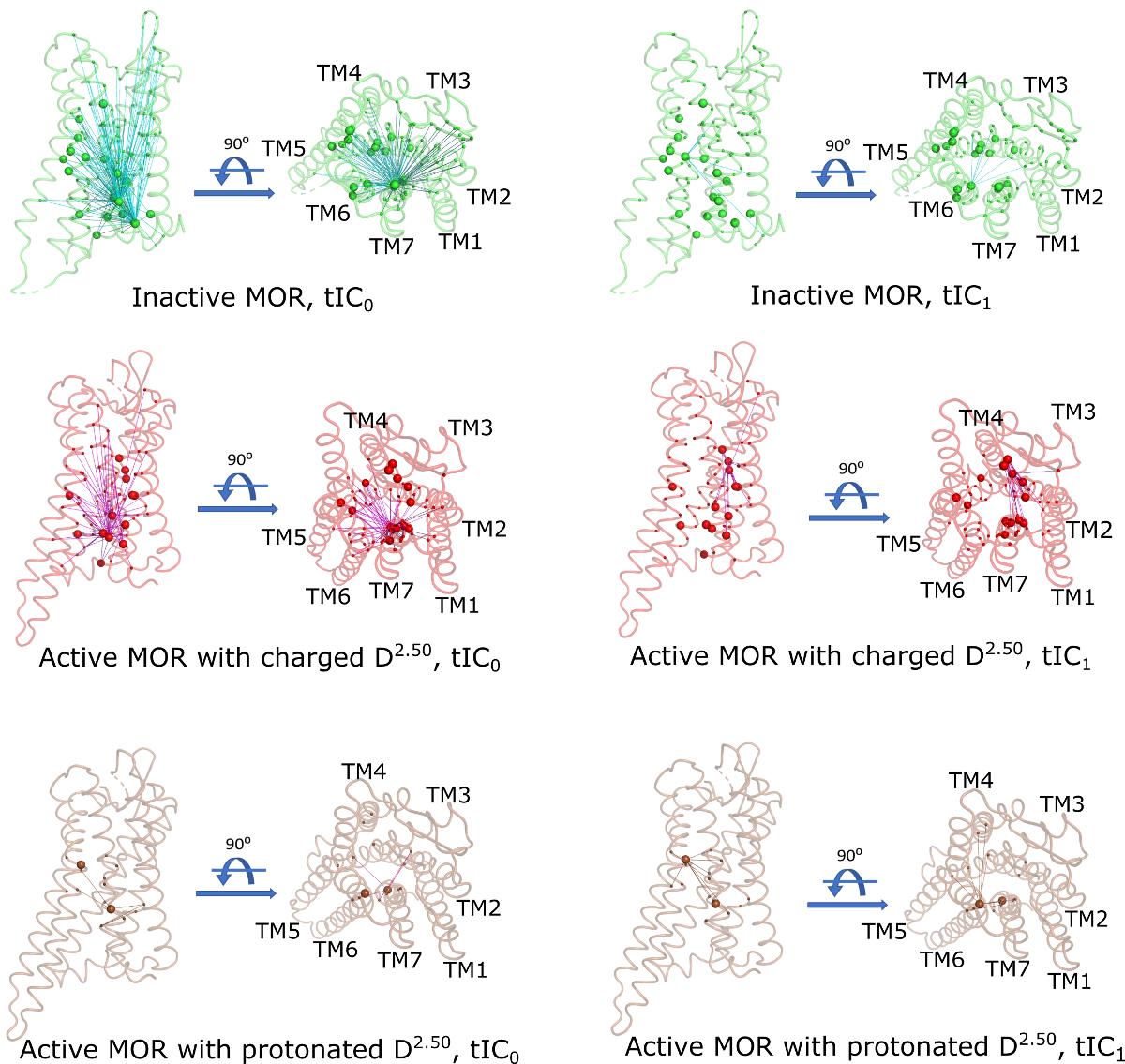

Supplement: S2 Fig — (TIFF) [file pcbi.1006689.s008.tiff]

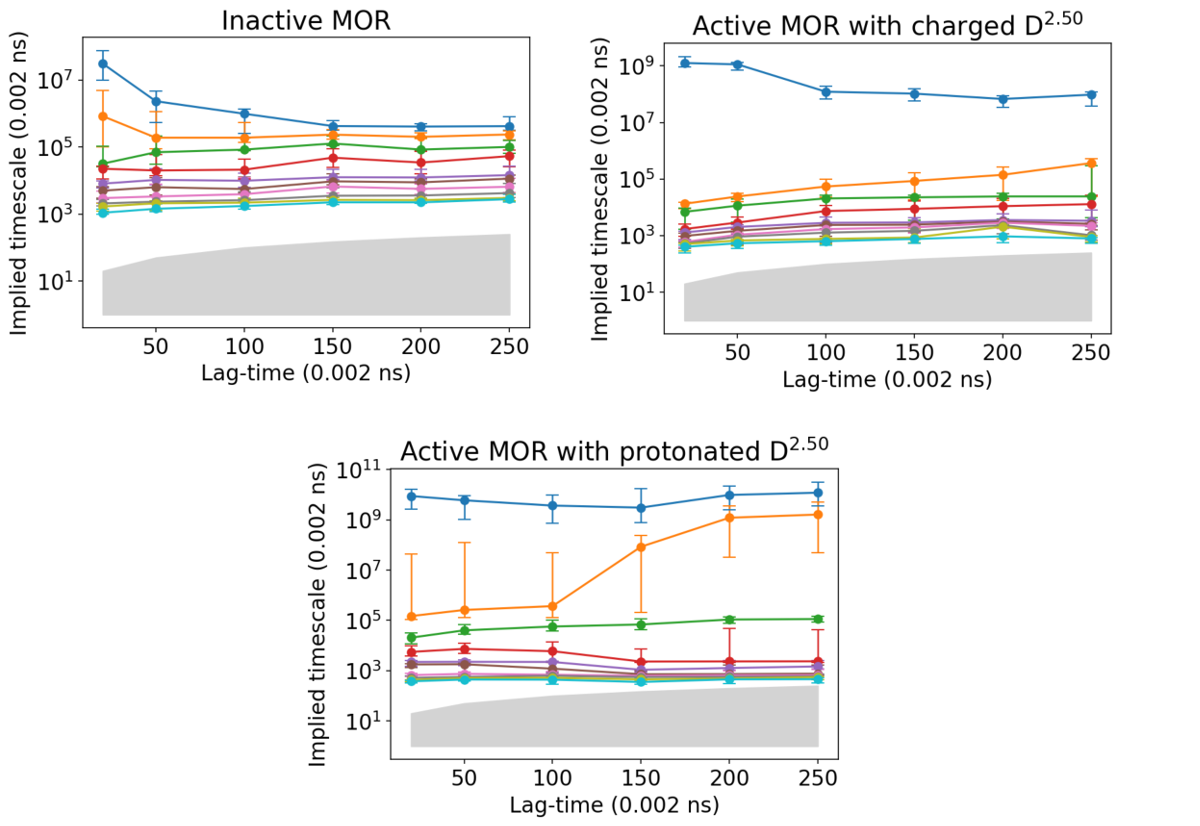

Supplement: S3 Fig — The data points correspond to the median estimated from all bootstraps samples and the full sample. The upper and lower error bars represent the differences between the median and 1st and 3rd quantiles, respectively. (TIFF) [file pcbi.1006689.s009.tiff]

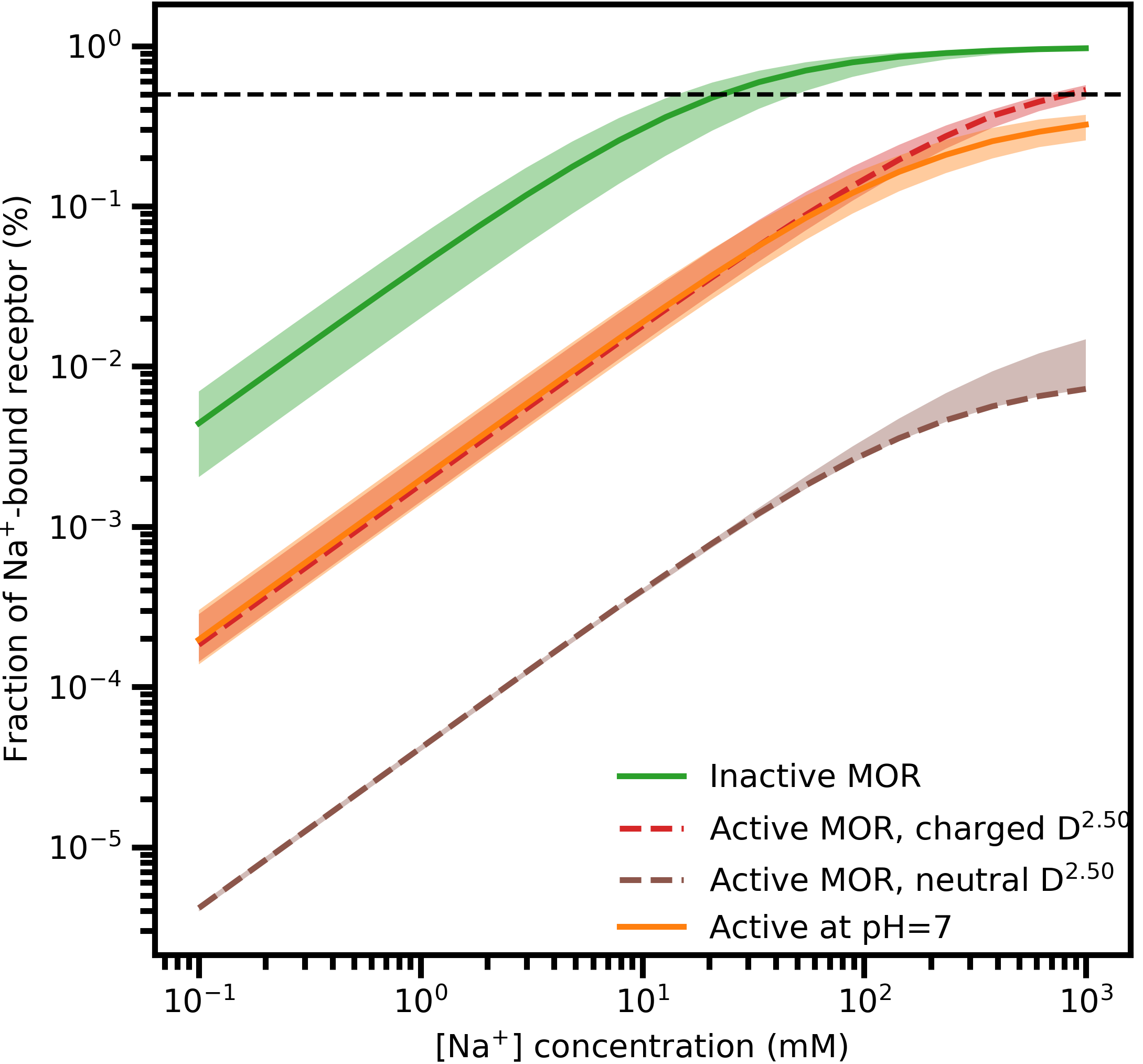

Supplement: S4 Fig — A dashed black line indicates 50% occupation probability that intersects the curves at the corresponding binding affinity values. The errors obtained from the bootstrap samples are shown as transparent colored bands around the lines. (TIFF) [file pcbi.1006689.s010.tiff]

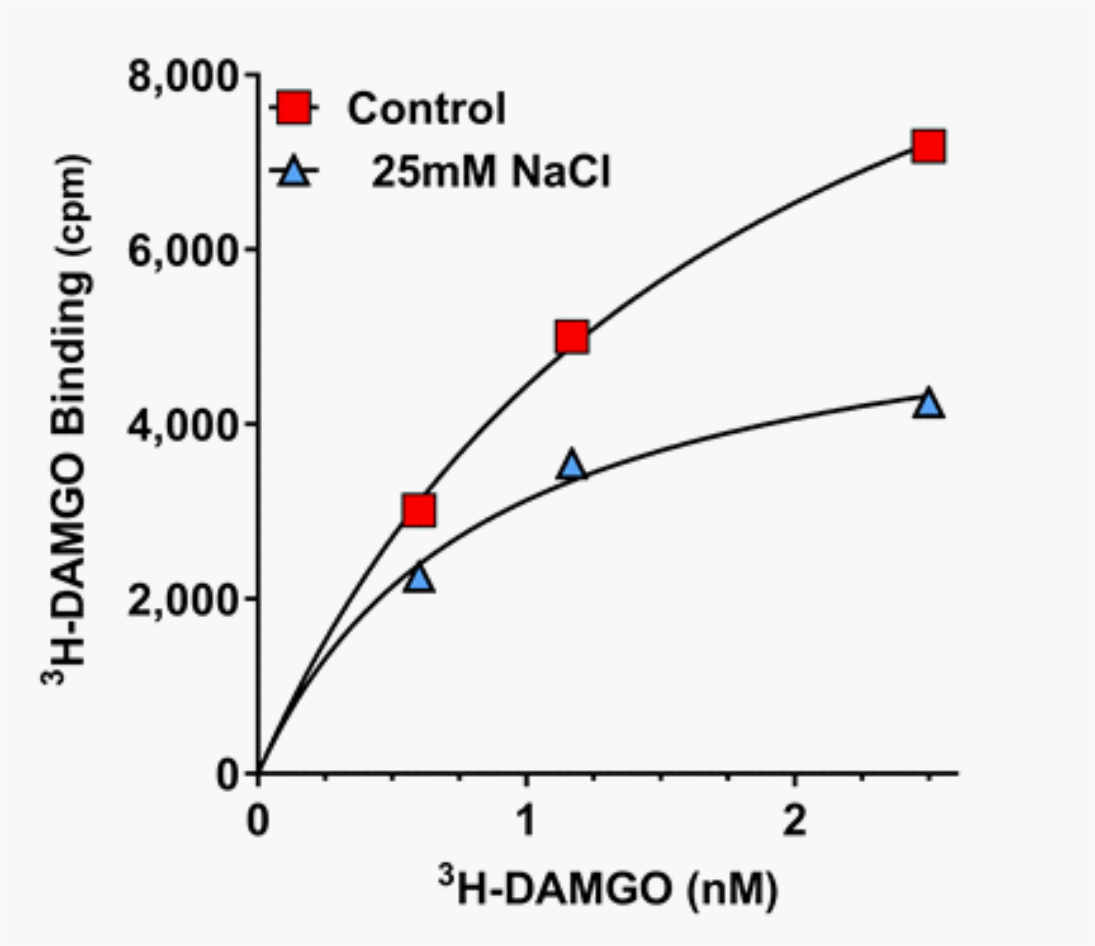

Supplement: S5 Fig — Results are the means ± s.e.m. of three independent replications. Nonlinear regression analysis of the curves is indicated by the solid lines. KD values for control and NaCl groups (1.78 and 0.87 nM, respectively) were similar while Bmax values revealed a decrease in the number of sites of 50% (333 fmol/mg protein to 156 fmol/mg proteins). (TIFF) [file pcbi.1006689.s011.tiff]
